# Preparation and Characterization of Lutein Co-Amorphous Formulation with Enhanced Solubility and Dissolution

**DOI:** 10.3390/foods13132029

**Published:** 2024-06-26

**Authors:** Xuening Song, Yingting Luo, Wenduo Zhao, Simiao Liu, Yuzhuo Wang, Hao Zhang

**Affiliations:** 1 College of Food Science and Nutritional Engineering, China Agricultural University, Beijing 100083, China; 2020306100616@cau.edu.cn (X.S.); s20213061018@cau.edu.cn (Y.L.); 2022306100208@cau.edu.cn (W.Z.); s20233061224@cau.edu.cn (S.L.); wyz@cau.edu.cn (Y.W.); 2 Beijing Laboratory of Food Quality and Safety, Department of Nutrition and Health, China Agricultural University, Beijing 100091, China; 3 Food Laboratory of Zhongyuan, Luohe 462300, China

**Keywords:** lutein, sucralose, co-amorphous, solubility enhancement, rotary evaporation

## Abstract

Lutein is an oxygenated fat-soluble carotenoid and a functional compound with proven health benefits for the human body. Nevertheless, the poor water solubility and low oral bioavailability of lutein greatly limit its application. To address this, we developed an effective approach to enhance the water solubility of lutein through co-amorphous formulation. Specifically, the lutein-sucralose co-amorphous mixture was prepared at a molar ratio of 1:1 using ethanol and water as solvents by employing the solvent evaporation method, followed by solid-state characterization and dissolution testing conducted to assess the properties of the formulation. The X-ray diffraction pattern with an amorphous halo and the differential scanning calorimetry thermogram with no sharp melting peaks confirmed the formation of a binary co-amorphous system. Changes in peak shape, position, and intensity observed in the Fourier transform infrared spectroscopy spectrum revealed intermolecular interactions between lutein and sucralose molecules, while molecular dynamics simulations identified interaction sites between their hydroxyl groups. Additionally, dissolution testing demonstrated better dissolution performance of lutein in the co-amorphous form compared to pure lutein and physical mixture counterparts. Our findings present a novel strategy for improving the water solubility of lutein to make better use of it.

## 1. Introduction

Carotenoids are classified into two main groups, carotenes and xanthophylls, based on their chemical structures [1]. Lutein is a xanthophyll, an oxygenated fat-soluble carotenoid, and a functional compound known for its beneficial effects on human health [2]. In the human body, lutein serves multiple physiological functions, including the maintenance of healthy vision and the reduction in risks associated with eye ailments, such as age-related macular degeneration (AMD), cataracts, and retinitis pigmentosa [3]. Furthermore, research indicates the involvement of lutein in improving and maintaining cognitive function [4], promoting cardiovascular health, and exhibiting potential in combating several types of cancers, such as breast cancer, gastric cancer, colon cancer, and pancreatic cancers [5].

The human body lacks the enzymes necessary to synthesize lutein; thus, it must be obtained from dietary sources [6]. Lutein-rich foods include dark-green leafy vegetables like lettuce, spinach, parsley, and kale, as well as yellow fruits and vegetables like mangoes, papayas, pumpkins, and corn [7]. Despite the availability of various food sources rich in lutein, the inadequate intake of lutein remains common. For example, the average American diet typically provides 1–3 mg of lutein daily, while research suggests that an intake of approximately 6 mg/day is necessary to lower the risk of developing macular degeneration and cataracts. Although there is no established Dietary Reference Intake (DRI) for lutein, clinical trials have shown it to be safe for consumption at a daily dose of 18 mg, with no adverse effects observed. This suggests it can be consumed at three times the recommended amount of 6 mg/day without any apparent harm [1]. Therefore, the development and formulation of functional foods containing lutein are essential.

Digestive fluids are primarily composed of water, making the limited solubility of lutein in water a significant obstacle to its absorption in the gastrointestinal tract, thereby reducing its bioavailability [8]. Additionally, the low solubility of lutein in water restricts its usage in the food and pharmaceutical industries. Consequently, many strategies have been investigated to improve the solubility of lutein, including the development of liposomes, nanoparticles, emulsions, microcapsules, and solid dispersions [9,10]. However, these techniques often have some limitations or drawbacks, such as poor storage stability, organic solvent contamination, high costs of additives, and limited drug-loading capacity [11], making large-scale industrial production challenging. In response to these challenges, the co-amorphous system has become increasingly popular in recent years.

The co-amorphous system involves combining two or more small molecules to form a homogeneous single-phase amorphous system [12]. In general, these components are linked by non-covalent interactions, such as the π-π stacking interaction, ionic bonding interaction, and hydrogen bonding interaction, or without any molecular interactions [13]. In recent years, a variety of co-amorphous systems have been reported to show enhanced solubility and improved dissolution performance. For example, Shayanfar et al. discovered that the water solubility of atorvastatin calcium-nicotinamide was 2-fold higher than that of pure atorvastatin calcium [14]. Samipillai et al. found that the dissolution rates of dasatinib-saccharin and olanzapine-saccharin were enhanced in phosphate-buffered saline (PBS, pH 7.2) at 37 °C, with enhancements of 20–30 times compared to the pure drugs [15]. Hu et al. reported that the solubility of tranilast-matrine in both water and PBS (pH 6.8) was over 100 times higher than that of crystalline tranilast, with significantly increased release rates [16]. However, at present, the co-amorphous system is mainly used in the pharmaceutical field, with limited utilization in the health products sector.

Sucralose, also known as trichlorogalactosucrose and TGS, is an extensively used non-nutritive and non-caloric artificial sweetener [17]. It is obtained by selectively replacing three hydroxyl groups in the sucrose molecule with three chlorine atoms. It is a hydrophilic compound exhibiting high water solubility (>25 mg/mL at 22 °C) due to the presence of five hydroxyl groups. Furthermore, sucralose is stable across a range of pH and temperatures, making it ideal for use in food production and processing [18]. Glucose, a simple sugar widely found in nature, is commonly used in candies, beverages, bakery products, and other foods. It plays a crucial role in the human body by providing energy for metabolism and the normal functioning of various organs. Fructose, an isomer of glucose, is the sweetest natural sweetener and is abundant in soft drinks and other foods. Lactitol, a sugar alcohol produced by the catalytic hydrogenation of lactose, is typically used to provide low-calorie sweetness and is widely utilized in formulating bakery, chocolate, and chewing gum [19]. Erythritol, a low-calorie natural sweetener, is commonly used in candies, beverages, baked goods, and dairy products. Furthermore, due to its antioxidant properties and glycolipid metabolic effects, it can also serve as an important ingredient in functional foods and health products [20]. While several studies have explored the use of small-molecule substances such as saccharin, amino acids, and citric acid as co-formers to improve the stability of co-amorphous systems [21], there is a dearth of research on co-amorphous systems consisting of lutein and these five small-molecule sweeteners. Hence, using these sweeteners as co-formers in co-amorphous systems remains largely unexplored.

Therefore, we focused on a novel co-amorphous system composed of lutein and sweeteners (sucralose, glucose, fructose, lactitol, and erythritol) prepared using rotary evaporation in this study. Various analytical techniques, including X-ray diffraction (XRD), differential scanning calorimetry (DSC), and Fourier transform infrared spectroscopy (FTIR), were employed to characterize the system. Molecular dynamics (MD) simulation was used to gain insights into the interaction sites of specific functional groups. Finally, the dissolution performance of the prepared co-amorphous mixture was evaluated through an in-vitro dissolution test.

## 2. Materials and Methods

### 2.1. Materials

Lutein was procured from the Shanghai Aladdin Bio-Chem Technology Co., Ltd. (Shanghai, China). Food-grade sucralose was purchased from the Zhejiang Yinuo Biotechnology Co., Ltd. (Hangzhou, China). Food-grade glucose, fructose, lactitol, and erythritol were acquired from the Shandong Pingju Biotechnology Co., Ltd. (Jining, China). Anhydrous ethanol (≥99.5%) was provided by the Tianjin Zhiyuan Chemical Reagent Co., Ltd. (Tianjin, China). Tween-80 was from the Hebei Yuanle Biotechnology Co., Ltd. (Shijiazhuang, China). Spectroscopic-grade potassium bromide (KBr) was obtained from the Shanghai Macklin Biochemical Co., Ltd. (Shanghai, China). Chromatographic-grade methanol and acetonitrile were supplied by the Beijing Mreda Technology Co., Ltd. (Beijing, China).

### 2.2. Preparation of Co-Amorphous Mixtures

The co-amorphous mixtures containing various sweeteners (sucralose, glucose, fructose, lactitol, and erythritol) as co-formers were prepared using the solvent evaporation method at a 1:1 molar ratio. Specifically, 67.5 mg of lutein was dissolved in 41.5 mL of anhydrous ethanol, while the co-former (47.2 mg of sucralose, 21.4 mg of glucose, 21.4 mg of fructose, 40.9 mg of lactitol, or 14.5 mg of erythritol) was dissolved in 3.5 mL of water. Subsequently, these two solutions were mixed and subjected to rotary evaporation until dryness at 60 °C and −0.09 MPa. To eliminate any residual solvent, the resulting product was further dried in a vacuum-drying oven at 45 °C for 12 h. Additionally, a physical mixture was prepared by uniformly blending the two powders at a molar ratio of 1:1 without any other processing or treatment. The prepared mixtures were stored at 4 °C until further experimentation.

### 2.3. Solubility Study

The solubility of the samples was determined by adding an excess amount of the sample to 10 mL of water and placing it in a shaking water bath at 25 ± 0.5 °C for 24 h. Afterward, the supernatant was diluted with three-fold ethanol and filtered through a 0.45 µm organic membrane before high-performance liquid chromatography (HPLC) analysis (Shimadzu, Kyoto, Japan). All solubility experiments were conducted in triplicate. The chromatographic conditions were set as follows: chromatographic column, Agela Technologies C18 (5 μm, 4.6 × 250 mm); mobile phase, methanol: ultrapure water: acetonitrile (*v*/*v*/*v* = 9/7/84); column temperature, 35 °C; flow rate, 1.0 mL/min; injection volume, 20 μL; detection wavelength, 445 nm [22].

To construct the lutein standard curve, lutein was accurately weighed and dissolved in anhydrous ethanol to prepare a mother liquor with a concentration of 10 μg/mL. From this mother liquor, a series of standard solutions of 0.05, 0.1, 0.25, 0.5, and 1 μg/mL were prepared by diluting with anhydrous ethanol and water. The solvent composition for each standard solution remained consistent, with a volume ratio of anhydrous ethanol to water equal to 3:1. Using the aforementioned chromatographic conditions, a standard curve was constructed with concentration as the abscissa and peak area as the ordinate. The linear regression equation of the standard curve was y = 157,146x − 6231.7 (R^2^ = 0.9991). Based on the corresponding standard curve and the peak area of the sample to be measured, the concentration of lutein in the sample in water could be obtained.

### 2.4. X-ray Diffraction (XRD)

The XRD patterns were obtained using an X-ray diffractometer equipped with a Cu Kα radiation source (Philips X’PERT MPD, Malvern Panalytical Inc., Westborough, MA, USA). The operating parameters were set to a working voltage of 36 kV and a current of 20 mA. Sample scanning was conducted in continuous mode over a range of 5° to 50° (2θ) at a step size of 0.02° and a scanning speed of 4°/min [23].

### 2.5. Differential Scanning Calorimetry (DSC)

DSC curves were recorded using a thermal analyzer system (DSC−60, SHIMADZU, Kyoto, Japan). Accurately weighed samples (3 to 5 mg) were placed into aluminum pans and sealed tightly. Meanwhile, an empty, sealed standard aluminum pan was used as a reference for instrument calibration. The pans were then heated from 30 to 250 °C in a continuous nitrogen purge of 50 mL/min, with a heating rate of 10 °C/min [24].

### 2.6. Fourier Transform Infrared Spectroscopy (FTIR)

FTIR measurements were conducted on an FTIR spectrometer (Spectrum 100−Spectrum, PerkinElmer Inc., Waltham, MA, USA). Approximately 2 mg of each dried sample was uniformly mixed with 200 mg of KBr by grinding in an agate mortar under infrared light and compressed into tablets before measurement. Spectral data were collected by performing 32 scans in transmission mode, covering the wavenumber range from 4000 to 400 cm^−1^ with a resolution of 4 cm^−1^ [16]. The FTIR spectrum of pure KBr served as the background of all samples.

### 2.7. Molecular Dynamics (MD) Simulation

The initial 3D molecular structures of lutein (PubChem CID: 5281243) and sucralose (PubChem CID: 71485) were retrieved from the PubChem database (https://pubchem.ncbi.nlm.nih.gov/, accessed on 25 January 2024). Subsequently, geometric optimization was carried out over 10,000 times using the Forcite code (Materials Studio 2020). Employing the Amorphous cell module, a lutein-sucralose co-amorphous system (molar ratio = 1:1) with a number ratio of 30/30 was assembled in a box with periodic boundary conditions (PBC). The dimensions of the box were set to 36.4 × 36.4 × 36.4 Å, and the density of the lutein-sucralose system was estimated based on the densities of pure lutein and sucralose. Subsequently, simulations in both the isothermal–isobaric ensemble (NPT) and canonical ensemble (NVT) were performed with a time step of 1.0 fs using the COMPASS force field [25]. Then, radial distribution function (RDF) analysis was performed using equilibration configuration.

### 2.8. Dissolution Test

The supersaturated dissolution test was conducted using a constant temperature magnetic stirrer (HJ-6B, Jintan Science Analysis Instrument Co., Ltd., (Changzhou, China). Initially, accurately weighed crystalline lutein, lutein-sucralose physical mixture, and lutein-sucralose co-amorphous mixture, each equivalent to 100 mg of lutein, were separately added to 100 mL of 0.1% Tween-80 solution. The rotation speed was maintained at 100 rpm, and the temperature of the dissolution medium was held constant at 37 ± 0.5 °C. At predefined time intervals (0.25, 0.5, 0.75, 1, 1.5, 2, 4, 6, 8 h), approximately 1 mL of each sample was withdrawn and filtered through a 0.45 μm filter to remove undissolved material [26]. Subsequently, the filtered samples were diluted with three-fold anhydrous ethanol and assayed for lutein concentration using the HPLC method. The dissolution experiments were performed in triplicate to ensure accuracy and reproducibility.

### 2.9. Statistical Analysis

Data analysis and graphical representation were performed using OriginPro 2022 software (OriginLab, Northampton, MA, USA) and SPSS 26.0 (IBM Co., Armonk, NY, USA). Experimental results were expressed as the mean ± standard deviation (SD), with each group comprising three samples (n = 3). Statistical analysis included one-way analysis of variance (ANOVA) followed by Tukey’s test for multiple comparisons. A *p*-value below 0.05 (*p* < 0.05) was considered statistically significant.

## 3. Results and Discussion

### 3.1. Co-Former Screening

**Figure 1:**
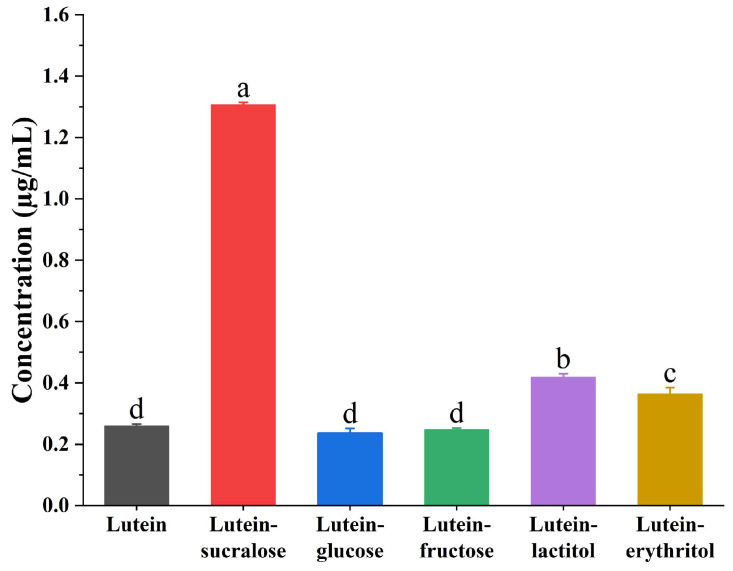
Saturated solubility of lutein and solid formulations of lutein-sucralose, lutein-glucose, lutein-fructose, lutein-lactitol, and lutein-erythritol in water. Different lowercase letters indicate statistically significant differences, *p* < 0.05.

The saturated solubility of lutein and solid formulations with various sweeteners (sucralose, glucose, fructose, lactitol, and erythritol) as co-formers was determined using HPLC, and the results are presented in Figure 1. Pure lutein exhibited a low solubility of approximately 0.26 μg/mL in water, confirming its poor water solubility. Additionally, different co-formers had variable effects on the solubility of lutein. Solid formulations with glucose and fructose as co-formers showed no significant difference in solubility compared to pure lutein. This observation suggested that glucose or fructose may not have formed co-amorphous systems with lutein, resulting in lutein remaining in a poorly water-soluble crystalline state. In contrast, solid formulations with sucralose, lactitol, and erythritol as co-formers significantly increased the solubility of lutein compared to pure lutein (*p* < 0.05). Among them, the lutein-sucralose solid formulation exhibited a five-fold enhancement in solubility relative to pure lutein.

Intermolecular hydrogen bonding interactions were crucial in promoting the compatibility between active substances and co-formers, thereby maximizing the likelihood of forming single-phase and effective co-amorphous systems [27]. Sucralose, with its five hydrogen bond donors and eight hydrogen bond acceptors [18], was particularly conducive to forming hydrogen bonds with lutein, hence forming a lutein-sucralose co-amorphous mixture. The amorphous nature of lutein in lutein-sucralose solid formulation exhibited a higher solubility compared to pure lutein, attributable to the reduced energy barrier required for molecule disintegration [28]. Given that sucralose had the most significant impact on improving the solubility of lutein, the lutein-sucralose solid formulation was selected for further characterization.

### 3.2. Solid State Characterization

#### 3.2.1. X-ray Diffraction (XRD)

**Figure 2:**
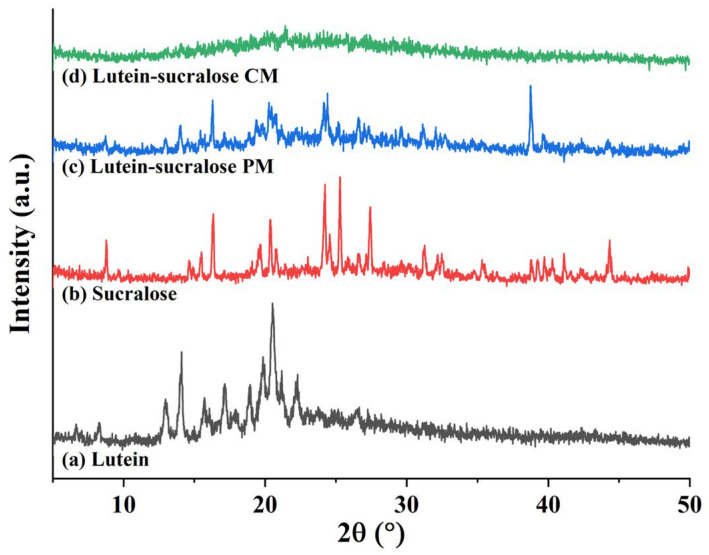
XRD patterns of (**a**) lutein, (**b**) sucralose, (**c**) lutein-sucralose physical mixture (PM), and (**d**) lutein-sucralose co-amorphous mixture (CM).

XRD is widely recognized as a gold standard method for characterizing amorphous solids. The appearance of an amorphous halo pattern in the diffraction graph is indicative of a decrease in crystallinity [29]. Wang et al. found that an amorphous halo was observed in the diffractogram for the investigated lacidipine-spironolactone co-amorphous system, indicating that the crystalline drugs were converted into an amorphous form in the system [21]. Figure 2 shows the XRD patterns of lutein, sucralose, the lutein-sucralose physical mixture, and the lutein-sucralose co-amorphous mixture. The lutein displayed strong characteristic diffraction peaks at 2θ angles of 12.96°, 14.1°, 15.72°, 17.16°, 18.94°, 19.86°, 20.52°, 21.18°, and 22.3°, consistent with previous literature reports [30], indicating its crystalline structure. Similarly, sucralose exhibited several distinct diffraction peaks within the range of 8° to 45°, especially at 2θ angles of 8.78°, 16.34°, 20.38°, 24.22°, 24.56°, 25.3°, and 27.44°. As expected, the diffraction pattern of the lutein-sucralose physical mixture resembled a superposition of their peaks, suggesting the existence of both substances in their crystalline forms. On the contrary, the lutein-sucralose solid formulation only exhibited a broad halo, with the absence of crystal diffraction peaks for both sucralose and lutein, signifying the formation of the lutein-sucralose co-amorphous mixture.

#### 3.2.2. Differential Scanning Calorimetry (DSC)

**Figure 3:**
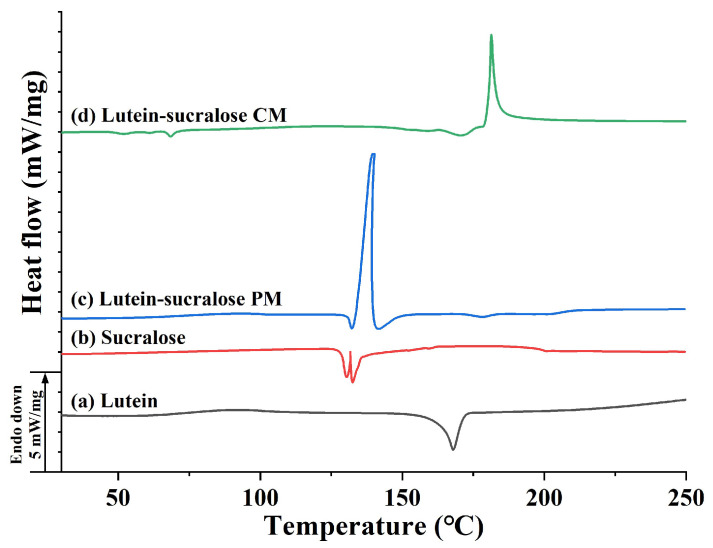
DSC thermograms of (**a**) lutein, (**b**) sucralose, (**c**) lutein-sucralose physical mixture (PM), and (**d**) lutein-sucralose co-amorphous mixture (CM).

DSC serves as an efficient method used for determining the thermodynamic properties of co-amorphous systems. Sharp endothermic peaks indicate the presence of a crystalline structure and an anhydrous state [31]. The DSC thermograms of lutein, sucralose, the lutein-sucralose physical mixture, and the lutein-sucralose co-amorphous mixture are presented in Figure 3. Lutein displayed a sharp endothermic peak at 168.0 °C, indicative of crystal melting [32]. Similarly, the endothermic peaks observed in the sucralose curve corresponded to melting effects. Regarding the physical mixture, it exhibited an endothermic peak at 132.2 °C, indicating its melting point and crystalline nature, alongside an intense exothermic peak at 140.0 °C caused by decomposition. In response to the phenomenon that the melting point and decomposition temperature of the physical mixture were shifted compared to pure components, we speculated that there may be interactions between lutein and sucralose molecules during the heating process, resulting in the formation of aggregates in situ [25]. Upon heating, the lutein-sucralose co-amorphous mixture showed two peaks, while the original melting peaks of lutein and sucralose disappeared completely. The low-intensity endothermic peak at 68.4 °C indicated sample dehydration, and the strong exothermic peak at 181.3 °C likely stemmed from thermal decomposition of the sample. Based on the DSC thermogram with no sharp melting peaks, coupled with the amorphous state evident in the XRD pattern, it was confirmed that a binary co-amorphous system was formed.

#### 3.2.3. Fourier Transform Infrared Spectroscopy (FTIR)

**Figure 4:**
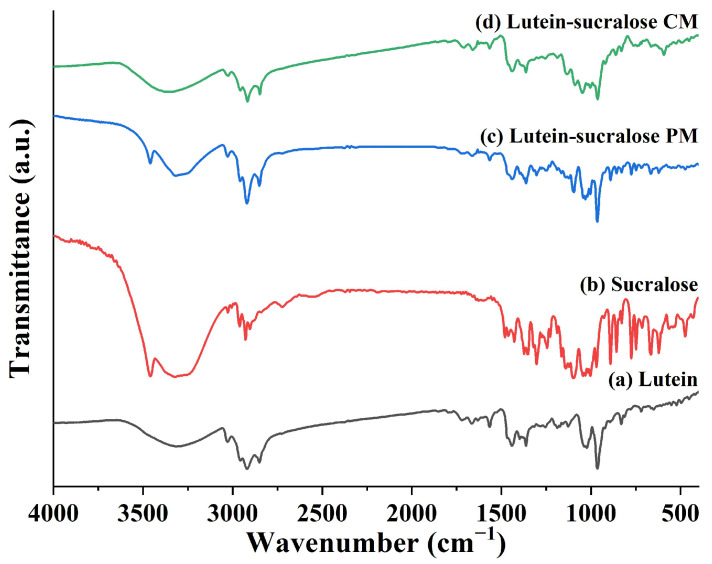
FTIR spectra of (**a**) lutein, (**b**) sucralose, (**c**) lutein-sucralose physical mixture (PM), and (**d**) lutein-sucralose co-amorphous mixture (CM).

FTIR is an effective approach for studying molecular interactions in co-amorphous systems. The changes in peak shape, position, and intensity indicate the presence of intermolecular interactions [33]. Hu et al. found that the stretching vibration peaks in the matrine-resveratrol co-amorphous mixture, specifically the O-H peak of resveratrol and the C=O peak of matrine, broadened and shifted noticeably, indicating possible intermolecular interactions between matrine and resveratrol [34]. Figure 4 shows the FTIR spectra of lutein, sucralose, the lutein-sucralose physical mixture, and the lutein-sucralose co-amorphous mixture. The wide band ranging from 3100 cm^−1^ to 3500 cm^−1^ corresponded to the stretching vibration of hydroxyl groups [35]. In the FTIR spectrum of lutein, the wide band at 3315 cm^−1^ arose from O-H stretching vibrations. The peaks observed at 2920 cm^−1^ and 2852 cm^−1^ corresponded to asymmetric and symmetric stretching vibrations of CH_2_ and CH_3_. The observed peak at 1440 cm^−1^ was related to CH_2_ scissoring, while the one at 1363 cm^−1^ was linked to dimethyl group splitting. Furthermore, the prominent peak at 964 cm^−1^ was designated for the out-of-plane deformation of trans-conjugated alkene -CH=CH- [32]. As for sucralose, the bands noticed at 3459 cm^−1^ and 3320 cm^−1^ were associated with O–H stretching modes [36]. The multiple peaks within the 2800–3000 cm^−1^ range corresponded to symmetric and asymmetric stretching of C-H, and the various bands within 650–1200 cm^−1^ were associated with vibration modes of the C-O, C-C, and C-O-H groups [37]. The FTIR spectrum of the physical mixture was a simple superposition of lutein and sucralose, retaining the characteristic peaks of both pure components. However, compared to the physical mixture, the absorption peak of O-H groups in the lutein-sucralose co-amorphous mixture notably shifted to 3356 cm^−1^, slightly weakening, and the peak shape became broader and less sharp. Overall, intermolecular interactions likely existed between lutein and sucralose molecules.

#### 3.2.4. Molecular Dynamics (MD) Simulation

**Figure 5:**
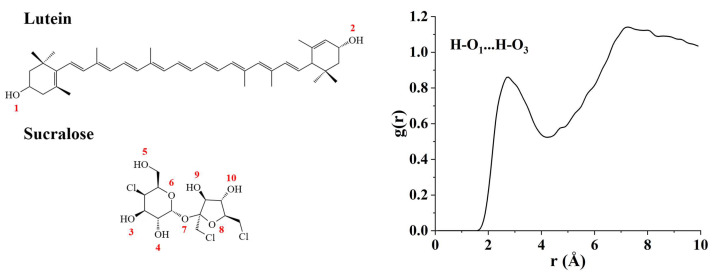
RDF analysis of the lutein-sucralose co-amorphous mixture.

FTIR results indicated the presence of molecular-level interactions between lutein and sucralose, although they were insufficient to determine the specific interaction sites of functional groups. MD simulation is a powerful tool to complement experimental results, offering deeper insights into the intermolecular interactions found within co-amorphous systems. The potential interaction sites in the lutein-sucralose co-amorphous mixture were further investigated through the analysis of radial distribution functions (RDF). The relationship between atomic density g(r) and interatomic distance (r) can be characterized based on RDF. Typically, the g(r) peak associated with hydrogen bonds occurs between 1.5 Å and 3.5 Å [38]. The chemical structural formulas of lutein and sucralose molecules and the RDF curve of the lutein-sucralose co-amorphous mixture are presented in Figure 5. RDF analysis was conducted between the atoms of lutein and sucralose, revealing a pair of interaction sites with a g(r) peak appearing within 3.5 Å. Specifically, the oxygen atom at O_1_ of lutein, concerning the distance of the hydrogen atom at O_3_-H of sucralose, exhibited a g(r) peak at 2.7 Å, indicating O_1_ and O_3_-H tended to form intermolecular hydrogen bonds. Therefore, the lutein and sucralose molecules likely primarily interacted via H-O_1_…H-O_3_ hydrogen bonds, with O_3_-H of sucralose acting as hydrogen bond donors and O_1_ of lutein acting as hydrogen bond acceptors.

### 3.3. Dissolution Test

**Figure 6:**
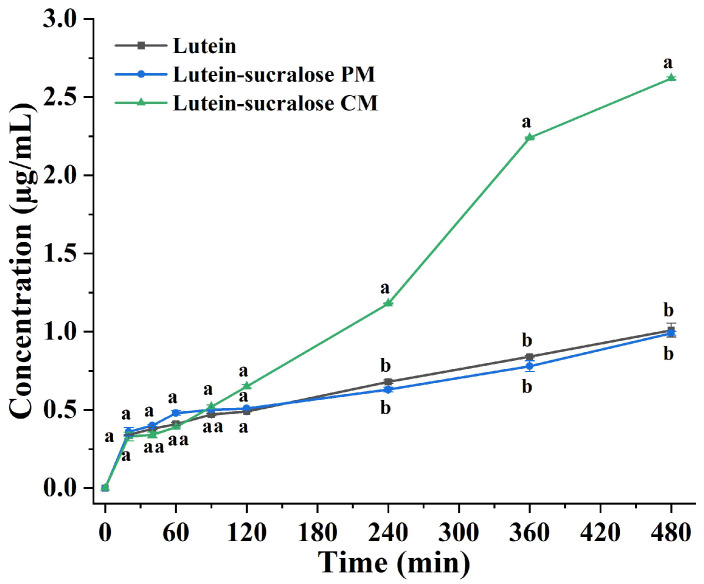
Dissolution profiles of lutein, lutein-sucralose physical mixture (PM), and lutein-sucralose co-amorphous mixture (CM) in 0.1% Tween-80 aqueous solution at 37 ± 0.5 °C. Different lowercase letters indicate statistically significant differences at each time interval, *p* < 0.05.

Dissolution plays a crucial role in formulation development, acting as the rate-limiting step for bioavailability [39]. The dissolution profiles of pure lutein, the lutein-sucralose physical mixture, and the lutein-sucralose co-amorphous mixture were determined, and the results are presented in Figure 6. Lutein and the lutein-sucralose physical mixture exhibited similar dissolution performance, suggesting that the simple mixing of the two components had minimal impact on lutein dissolution. However, the dissolution profile of the lutein-sucralose co-amorphous mixture differed significantly from those of the other two groups. Notably, after 120 min of dissolution, the concentration of lutein in the co-amorphous form began to exhibit significant differences (*p* < 0.05). The dissolution concentration of lutein in the co-amorphous form was nearly three times that of the other groups at 360 min. Subsequently, the dissolution rate gradually slowed, and the concentration tended to reach equilibrium. The observed increase in dissolution in co-amorphous systems was often attributed to the “spring and parachute mechanism” [13]. Amorphous lutein lacked long-range crystallographic order, resulting in higher free energy and lower dissolution lattice energy, thus facilitating dissolution [40]. Moreover, hydrogen-bonding interactions between lutein and sucralose impeded nucleation and crystal growth, thereby preventing the recrystallization of lutein [41]. Consequently, the lutein-sucralose co-amorphous mixture exhibited increased supersaturation levels throughout the dissolution study, which is beneficial for enhancing gastrointestinal absorption to a certain extent [29].

## 4. Conclusions

In this study, we prepared solid formulations of lutein with sucralose, glucose, fructose, lactitol, and erythritol using the solvent evaporation method. Among them, the lutein-sucralose solid formulation exhibited a remarkable five-fold increase in solubility compared to pure lutein, making it the most promising combination for solubility enhancement. Characterization techniques such as XRD patterns and DSC thermograms confirmed the formation of a binary co-amorphous mixture, while FTIR spectra revealed intermolecular interactions between lutein and sucralose molecules. Molecular dynamics simulation provided insights into the interaction sites between the hydroxyl groups of lutein and sucralose. Furthermore, dissolution testing demonstrated the superior performance of lutein in the co-amorphous form compared to both pure lutein and the physical mixture. Compared with existing studies on improving the solubility of lutein, such as liposomes, nanoparticles, and emulsions, the lutein-sucralose co-amorphous mixture has the advantages of high loading capacity, low costs of additives, and no organic solvent contamination, making large-scale industrial production possible. This underscores the potential of the lutein-sucralose co-amorphous mixture to be formulated into a wide range of food items, like functional foods and nutraceutical products.

## Data Availability

The data presented in this study are available on request from the corresponding author. The data are not publicly available to preserve the scientific integrity of the research methodology.
